# Diabetic Kidney Disease Progression Alleviated in Mice by ALKBH5‐Mediated UC‐MSCs‐Derived Exosomes That Inhibit TRAF6 m6A Modification and Promote M2 Macrophage Polarisation

**DOI:** 10.1002/edm2.70131

**Published:** 2026-01-13

**Authors:** Lei Li, Hongmei Liu, Huanhuan Wang, Yu Mao, Lige Song, Zhiqiang Kang

**Affiliations:** ^1^ Department of Endocrinology Zhengzhou Central Hospital Affiliated to Zhengzhou University Zhengzhou City Henan Province China

**Keywords:** ALKBH5, diabetic kidney disease, exosomes, TRAF6, umbilical cord mesenchymal stem cells

## Abstract

**Background:**

Diabetic kidney disease (DKD) is a major diabetes complication with limited treatment options. Exosomes (Exo) from umbilical cord mesenchymal stem cells (UC‐MSCs) have shown therapeutic promise. The role of alkylation repair homologue protein 5 (ALKBH5)‐modified UC‐MSCs Exo in regulating macrophage polarisation and alleviating DKD is investigated.

**Methods:**

DKD‐associated inflammation was modelled by Lipopolysaccharide (LPS)/interferon‐gamma (IFN‐γ)‐stimulated RAW264.7 macrophages. RT‐qPCR and western blotting were employed to analyse mRNA and protein expression. Exosomes from ALKBH5‐modified UC‐MSCs were isolated and characterised. Macrophage polarisation (M1/M2) was assessed by flow cytometry, RT‐qPCR, and enzyme‐linked immunosorbent assay (ELISA). Tumor necrosis factor receptor‐associated factor 6 (TRAF6) N6‐methyladenosine (m6A) modification and expression were analysed via methylated RNA immunoprecipitation (MeRIP) and RNA immunoprecipitation (RIP) assays. The DKD model was established using spontaneous diabetic db/db mice. The renal function of mice was evaluated by ELISA and commercial assay kits. Hematoxylin–eosin (HE), periodic acid‐Schiff (PAS), and Masson's trichrome staining were performed to assess the renal histopathology of mice.

**Results:**

ALKBH5 overexpression promoted M2 and inhibited M1 macrophage polarisation. ALKBH5 downregulated TRAF6 via m6A demethylation. ALKBH5‐modified UC‐MSCs Exo enhanced M2 polarisation and suppressed M1 phenotype in vitro. In DKD mice, ALKBH5‐modified UC‐MSCs Exo mitigated renal injury. Moreover, these exosomes enhanced anti‐inflammatory responses and promoted M2 macrophage polarisation in DKD mice.

**Conclusion:**

ALKBH5‐modified UC‐MSCs Exo reduced TRAF6 expression by demethylating its m6A sites, promoting M2 macrophage polarisation and alleviating DKD progression. These findings suggested that ALKBH5‐modified UC‐MSCs Exo might represent a promising therapeutic approach for DKD.

## Graphical abstract

ALKBH5‐modified UC‐MSCs‐derived exosomes (OE‐ALKBH5 Exo) inhibit the expression of M1 markers (iNOS, IL‐1β, TNF‐α, CD86) and enhance the expression of M2 markers (IL‐10, Arg‐1, CD206) by inhibiting the m6A methylation of TRAF6 and reducing its protein level and stability, thereby promoting M2 polarisation and alleviating the progression of DKD.

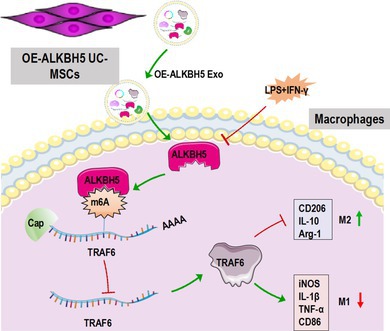

## 1 Introduction

Diabetic kidney disease (DKD) has emerged as a critical worldwide health concern, representing the most severe microvascular complication of diabetes mellitus and directly impairing renal function [1]. Hence, a deeper understanding of the molecular pathways involved in DKD progression is crucial for identifying new treatment strategies and developing more effective drug therapies.

Various immune and inflammatory cells are actively involved in the initiation and advancement of DKD. Among these, macrophages are key inflammatory mediators that infiltrate the kidney, contribute to the inflammatory microenvironment, and play a pivotal role in DKD‐associated inflammation [2]. Macrophages exhibit functional plasticity, displaying a dichotomy between pro‐inflammatory M1 and anti‐inflammatory M2 phenotypes [3]. The pro‐inflammatory M1 macrophage phenotype is induced by stimuli such as interferon‐γ (IFN‐γ), lipopolysaccharide (LPS), and tumour necrosis factor‐α (TNF‐α). Upon activation, M1 macrophages upregulate inducible nitric oxide synthase (iNOS) and secrete inflammatory mediators, including interleukin‐1β (IL‐1β), IL‐6, and TNF‐α [4]. In contrast, M2 macrophage activation is mediated by IL‐10 and IL‐4. M2 macrophages express arginase 1 (Arg1) and facilitate tissue repair through the release of anti‐inflammatory cytokines such as transforming growth factor‐β and IL‐10 [5]. Suppressing inflammatory signalling from M1 macrophages while promoting the secretion of anti‐inflammatory cytokines by M2 macrophages can help alleviate DKD [6]. Therefore, modulating the phenotypic transition between M1 and M2 macrophages could represent a promising therapeutic strategy for DKD.

Diverse cellular processes and biological functions are regulated by N6‐methyladenosine (m6A), the most abundant RNA methylation modification in mRNAs [7]. Notably, m6A modification critically regulates macrophage polarisation and function [8]. For instance, methyltransferase METTL14 deletion shifts microglia/macrophages from M1 to M2 phenotype and inhibits NLRP3 inflammasome activation, reducing brain injury after stroke [9]. As an m6A demethylase, Alkylation repair homologue protein 5 (ALKBH5) is involved in RNA metabolism, regulating its stability, transport, and various biological processes [10]. ALKBH5 inhibits gastric cancer cell invasion through m6A‐dependent regulation of PKMYT1 expression [11]. Moreover, CD133+ hepatocellular carcinoma‐derived exosomes have been reported to transfer ALKBH5 to THP‐1 cells, promoting M2 macrophage polarisation [12]. Nevertheless, the precise role of ALKBH5 in macrophage polarisation during DKD pathogenesis remains to be fully elucidated. Tumour necrosis factor receptor‐associated factor 6 (TRAF6) is a ubiquitin ligase that serves as a key binding partner for both the TNF superfamily and the toll/IL‐1 receptor superfamily [13]. TRAF6 has also been implicated as a therapeutic target for mitigating inflammatory injury in DKD. TRAF6 knockdown in the kidneys of diabetic mice can alleviate renal inflammation [14]. However, it remains unclear whether ALKBH5 mediates the m6A modification of TRAF6 to influence the progression of DKD.

Mesenchymal stem cells (MSCs) have gained increasing recognition in regenerative medicine due to their therapeutic potential. With advancements in cell‐based therapies, MSCs offer a potential strategy to combat DKD [15, 16]. It is well established that MSCs have the ability to self‐renew and differentiate into multiple cell types. Current evidence indicates that human umbilical cord (HUC) is an optimal MSC source for clinical applications, primarily due to its low immunogenicity. The therapeutic potential of UC‐MSCs arises from their dual capabilities of indefinite proliferation and differentiation into multiple cell types. Their low immunogenicity makes UC‐MSCs particularly suitable for clinical applications [17]. Emerging evidence suggests that exosomes derived from MSCs (MSCs Exo) primarily contribute to MSC‐mediated tissue repair through paracrine mechanisms [18, 19]. MSCs Exo have gained significant attention as a promising cell‐free therapeutic approach with inherent immunologic safety [20]. However, the functions of many components in natural MSCs Exo remain unclear, leading to poor therapeutic specificity and the potential for unexpected side effects, which limit their application in the treatment of DKD [21]. Consequently, modification of MSCs Exo through various strategies is warranted.

This study aimed to elucidate whether ALKBH5‐modified UC‐MSCs Exo influenced macrophages by modulating the m6A modification of TRAF6, thereby affecting DKD progression, and to provide mechanistic insights that may inform future DKD therapeutic research.

## 2 Materials and Methods

### 2.1 Cell Culture and Treatment

UC‐MSCs and RAW 264.7 cells were obtained from Procell (Wuhan, China). UC‐MSCs were maintained in serum‐free stem cell culture medium (Lonza, Walkersville, MD, USA) at 37°C and 5% CO_2_. The incubator atmosphere consisted of 5% CO_2_ and 95% ambient air (approximately 78% nitrogen and 21% oxygen), with relative humidity maintained at > 95%. The isolation of UC‐MSCs was performed as previously described [22]. The phenotype of UC‐MSCs was characterised using flow cytometry with antibodies against CD34 (1:200, Abcam, Cambridge, UK), CD45 (1:20, Abcam), CD90 (1:300, Abcam), and CD105 (1:500, Abcam). RAW264.7 cells were cultured in Dulbecco's modified Eagle medium (DMEM, Invitrogen, Carlsbad, CA, USA) supplemented with 10% fetal bovine serum (FBS; Gibco, Carlsbad, CA, USA) under the same incubation conditions. To simulate the inflammatory microenvironment of DKD, RAW 264.7 cells were stimulated with LPS (100 ng/mL) and IFN‐γ (30 ng/mL). Cells were incubated at 37°C with 5% CO_2_ for 24 h before being used for subsequent analyses.

### 2.2 Cell Transfection

Small interfering RNA (siRNA) targeting ALKBH5 (si‐ALKBH5), TRAF6 (si‐TRAF6) and non‐targeting control siRNA (si‐NC) were synthesised by GenePharma (Shanghai, China). For overexpression, full‐length ALKBH5 and TRAF6 coding sequences were inserted into pcDNA3.1 vectors (GenePharma) to generate OE‐ALKBH5 and OE‐TRAF6, with empty vector as control. Cells were transfected employing Lipofectamine 3000 (Invitrogen) as the transfection reagent.

### 2.3 Real‐Time Quantitative Polymerase Chain Reaction (RT‐qPCR)

Cellular RNA extraction was performed with TRIzol reagent (Invitrogen), followed by cDNA synthesis using NovoScript Plus All‐in‐one 1st Strand cDNA Synthesis SuperMix (Novoprotein, Shanghai, China). RT‐qPCR analysis was conducted on a Light Cycler 96 system with NovoStart SYBR qPCR SuperMix Plus (Novoprotein). Gene expression levels were normalised to GAPDH and quantified via the 2^−ΔΔCT^ method, analysing ALKBH5, TRAF6, M1 markers (IL‐1β, TNF‐α, iNOS), and M2 markers (IL‐10, Arg‐1). The primers used for RT‐qPCR could be found in Table S1.

### 2.4 Western Blotting

Cells were lysed using RIPA buffer (Beyotime, Wuhan, China). Protein samples of equal quantity were resolved by SDS‐PAGE and electrotransferred onto polyvinylidene fluoride membranes (Beyotime). After blocking with 5% non‐fat milk (Beyotime), membranes were probed overnight at 4°C with primary antibodies targeting ALKBH5 (1:1000, ab195377, Abcam), TRAF6 (1:1000, ab40675, Abcam), TSG101 (1:1000, ab125011, Abcam), CD81 (1:1000, ab109201, Abcam), CD9 (1:1000, ab236630, Abcam), Calnexin (1:1000, ab22595, Abcam), p‐STAT1 (1:1000, 28977‐1‐AP, Proteintech, Wuhan, China), STAT1 (1:2000, 10144‐2‐AP, Proteintech), p‐p65 NF‐κB (1:2000, 82335‐1‐RR, Proteintech), p65 NF‐κB (1:5000, 80979‐1‐RR, Proteintech), GAPDH (1:1000, ab9485, Abcam). Then, membranes were incubated with HRP‐conjugated secondary antibody (1:1000, ab6721, Abcam) for detection. Protein signals were detected using an enhanced chemiluminescence kit (Beyotime) and quantified with NIH ImageJ software, with GAPDH serving as the loading control for normalisation.

### 2.5 Flow Cytometric Analysis

Macrophages from different groups were collected, digested with trypsin, and counted, with approximately 3–5 × 10^5^ cells per tube. Cells were centrifuged at 1000 rpm for 1 min, resuspended in sterile phosphate‐buffered saline (PBS, Beyotime) and washed once. After a second centrifugation, the pellet was resuspended in 100 μL of sterile PBS containing 10% FBS. Cells were stained with fluorescently labelled antibodies (FITC‐F4/80, APC‐CD206, or APC‐CD86; 1 μg each) at 4°C for 30 min. After centrifugation (1000 rpm, 1 min), cells underwent PBS washes to remove excess antibodies and were resuspended in 300 μL PBS with 10% FBS for F4/80/CD86/CD206 detection by flow cytometry. Each tube contained approximately 1 × 10^6^ macrophages, and three independent replicates were analysed for each group.

### 2.6 Inflammatory Factor Detection

IL‐1β, TNF‐α, and IL‐10 levels in cell culture supernatants and mouse serum were measured by enzyme‐linked immunosorbent assay (ELISA, Thermo Fisher Scientific) following the manufacturer's instructions.

### 2.7 RNA Immunoprecipitation (RIP) Assay

RIP assay was conducted using the Magna RIP Kit (Millipore, Burlington, MA, USA) following the manufacturer's instructions. Cellular lysates were prepared by incubating cells with RIP lysis buffer on ice for 5 min. Magnetic beads were pre‐coated with either ALKBH5‐specific antibodies or control IgG at room temperature for 30 min. Antibody‐bound beads were then incubated with cell lysates at 4°C overnight. The immunoprecipitated RNA‐protein complexes were subsequently digested with proteinase K in 10% SDS solution, followed by RNA isolation from the supernatant for RT‐qPCR analysis.

### 2.8 Methylated RNA Immunoprecipitation‐qPCR (MeRIP‐qPCR) Assay

The SRAMP database (http://www.cuilab.cn/sramp/) was used to predict m6A modification sites in TRAF6 mRNA. The MeRIP assay was performed with the EpiQuik CUT&RUN m6A RNA Enrichment Kit (Epigentek, Farmingdale, NY, USA) in accordance with the manufacturer's guidelines. For m6A immunoprecipitation, 10 μg RNA was incubated with m6A‐specific antibody‐coupled beads in immuno‐capture buffer (90 min, room temperature), using IgG as a negative control. Following fragmentation (4 min, cleavage enzyme mix/nuclease enhancer), beads were washed and digested with proteinase K (55°C, 15 min) after supernatant removal. Finally, RNA was purified and eluted in buffer for 5 min at room temperature. The enriched m6A‐modified RNA fragments were subsequently subjected to RT‐qPCR to assess the relative enrichment of TRAF6 mRNA.

### 2.9 RNA Stability Assay

The cells from each experimental group were cultured in medium containing 5 μg/mL actinomycin D (ActD, MCE, Monmouth Junction, NJ, USA) for different durations (0 h, 2 h, 4 h, and 8 h). At each designated time point, cells were harvested and subjected to RT‐qPCR analysis to determine the relative expression level of TRAF6 mRNA.

### 2.10 Isolation and Identification of Exosomes

UC‐MSCs transfected with either Vector or OE‐ALKBH5 constructs were selected based on optimal viability and cultured for 48 h. The cells were then incubated for an additional 24 h in fresh exosome‐depleted serum medium. Following incubation, 50 mL of conditioned medium was collected and processed for exosome isolation using the ExoQuick exosome precipitation solution (System Biosciences, Palo Alto, California, USA). The purified exosomes were subsequently used for characterisation and functional analyses. The isolated exosomes were named Vector Exo and OE‐ALKBH5 Exo, respectively. The morphology of the exosomes was examined using a transmission electron microscope (TEM). 20 μg of Vector Exo and OE‐ALKBH5 Exo protein samples were collected and analysed by western blotting to detect the expression of exosome marker proteins CD81, CD9, TSG101, and the exosome‐negative protein Calnexin. After incubating Vector Exo or OE‐ALKBH5 Exo with 10 μM Dil dye (Beyotime) at 37°C in the dark for 30 min, the exosomes were washed with PBS to remove unbound dye and then centrifuged at 10,000 g at 4°C for 70 min. The exosomes were subsequently co‐incubated with RAW264.7 cells at 37°C for 24 h. After washing the cells with PBS, they were fixed for 1 h and stained with DAPI (Beyotime) for 10 min. The exosome uptake was observed using a fluorescence microscope.

### 2.11 Animal Models and Treatments

A total of 21 male spontaneously diabetic mice (db/db, 6 weeks old) and 7 six‐week‐old male control mice (db/m) were purchased from SPF Biotech (Beijing, China). After a 7‐day acclimation in an SPF facility, the model group received a high‐fat, high‐sugar diet, while the control group had a standard diet. Bedding, water, and feed were regularly replenished. Mice were monitored for 10 weeks, with weekly blood glucose checks and periodic urine collection for microalbumin analysis. The DKD model was considered successfully established when model mice showed reduced activity, coarse fur, increased food and water intake, higher urine output, fasting blood glucose ≥ 16.7 mmol/L, and significantly elevated 24‐h urinary albumin excretion. The metabolic and physiological parameters of db/db mice before and after modelling are summarised in Table S2. The mice were randomly divided into four groups (*n* = 7 per group): db/m, db/db, db/db + Vector Exo, and db/db + OE‐ALKBH5 Exo. For 8 consecutive weeks, the db/m and db/db groups received weekly tail vein injections of 200 μL PBS, while the db/db + Vector Exo and db/db + OE‐ALKBH5 Exo groups were administered 100 μg [23] of exosomes via the same route. Metabolic evaluations were performed after an 8‐h fasting period, with blood glucose levels measured from tail vein samples using a glucometer. Additional venous blood samples were collected for blood urea nitrogen (BUN) and serum creatinine (Scr) analysis, and urine was obtained for albumin quantification. Mice were then sacrificed, and tissues were harvested. Renal histopathological assessment was performed on 4‐μm paraffin sections using haematoxylin and eosin (HE), periodic acid‐Schiff (PAS), and Masson's trichrome staining to examine glomerulosclerosis and renal injury. The staining procedures were performed following the protocols provided in the respective staining kits (Solarbio, Beijing, China). Immunohistochemistry (IHC) was also performed on kidney sections to evaluate the expression of ALKBH5 and TRAF6. Renal damage was assessed using semiquantitative scoring of glomerulosclerosis and tubulointerstitial injury (0–4 scale) [24]. Three sections per mouse were evaluated, with five randomly selected fields scored per section. The mean score of 15 fields was used per animal. The Experimental Animal Ethics Committee at Zhengzhou Central Hospital Affiliated to Zhengzhou University reviewed and approved all animal procedures. Male mice were selected because prior studies have demonstrated that males exhibit more severe renal damage in DKD, including greater glomerular injury and proteinuria, compared to females [25, 26]. In contrast, oestrogen is thought to play a protective role in females via modulation of oxidative stress and inflammation [27, 28]. Therefore, male mice were used in this mechanistic study to reduce variability and establish baseline efficacy.

### 2.12 Statistical Analysis

Results were expressed as mean ± standard deviation. The Shapiro–Wilk test was used to assess data normality. For normally distributed data, statistical analyses employed Student's *t*‐test (two‐tailed) for two‐group comparisons and one‐way ANOVA for multi‐group comparisons. Tukey's post hoc test was used for pairwise comparisons among multiple groups to control for Type I error. Specifically, the kidney damage scores were confirmed to be normally distributed and analysed using one‐way ANOVA followed by Tukey's HSD test. A *p*‐value < 0.05 was considered statistically significant.

## 3 Results

### 3.1 Overexpression of ALKBH5 Promotes M2 Polarisation and Inhibits M1 Polarisation in RAW264.7 Macrophages

**FIGURE 1:**
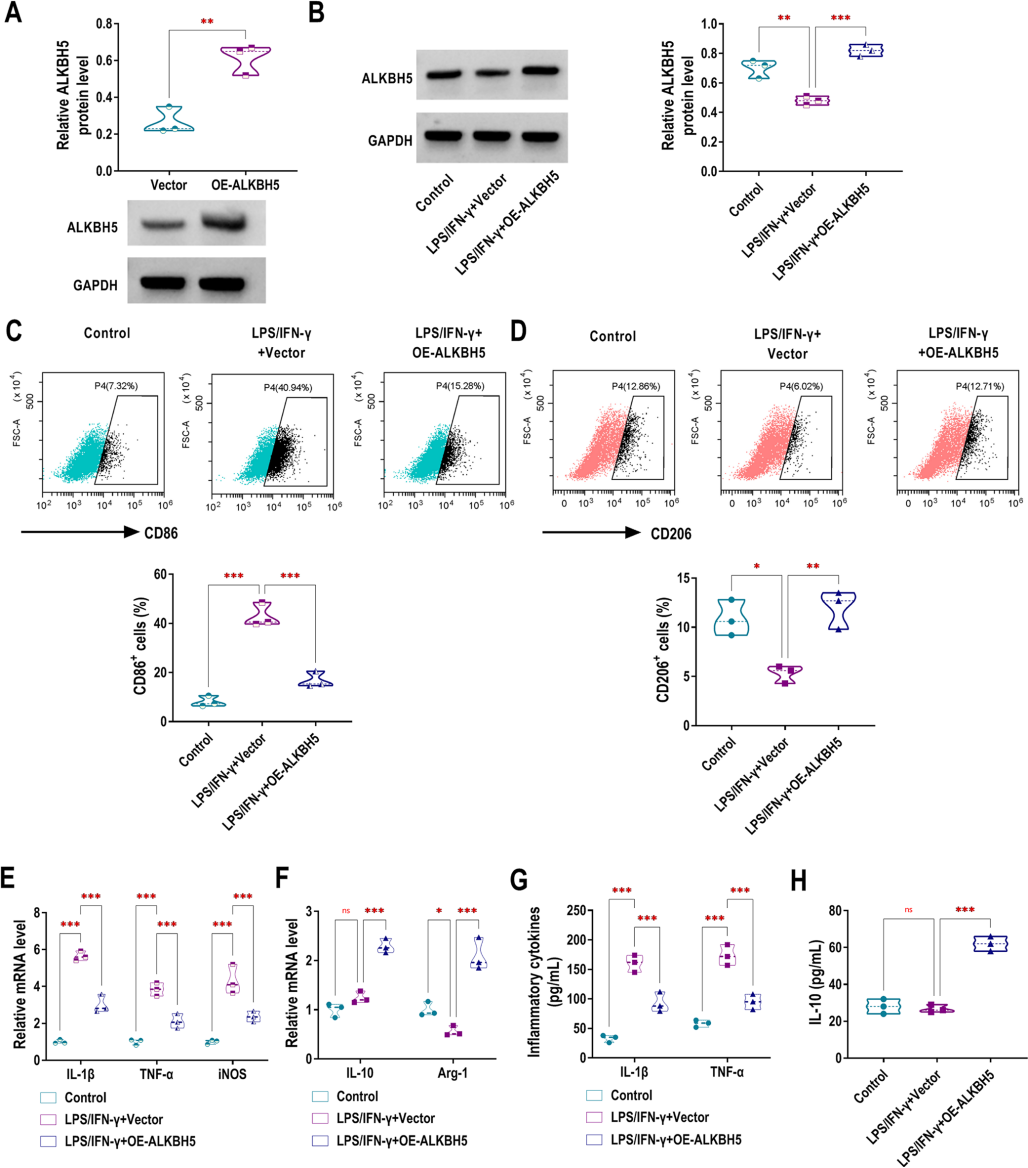
Overexpression of ALKBH5 promotes M2 polarisation and inhibits M1 polarisation in RAW264.7 macrophages. (A) The protein expression of ALKBH5 in RAW264.7 macrophages transfected with Vector or OE‐ALKBH5 was detected by western blotting. (B‐H) RAW264.7 cells stimulated by LPS/IFN‐γ were transfected with Vector or OE‐ALKBH5. (B) Western blotting was performed to examine ALKBH5 expression in cells. (C, D) Flow cytometry analysis was performed to assess the expression of the M1 macrophage marker CD86 and the M2 macrophage marker CD206. (E, F) RT‐qPCR was conducted to quantify the mRNA expression of M1‐associated inflammatory mediators (IL‐1β, TNF‐α, and iNOS) and M2 macrophage markers (IL‐10 and Arg‐1). (G, H) The levels of pro‐inflammatory cytokines (IL‐1β and TNF‐α) and the anti‐inflammatory cytokine (IL‐10) in the cell culture supernatant were measured using ELISA. ns, no significance, *p < 0.05, **p < 0.01, ***p < 0.001.

Initially, we overexpressed ALKBH5 in RAW264.7 macrophages, and western blotting results demonstrated the successful overexpression efficiency of OE‐ALKBH5 (Figure 1A). To construct an inflammatory cell model, we used LPS/IFN‐γ to stimulate RAW264.7 cells in vitro. LPS/IFN‐γ stimulation significantly inhibited the protein expression of ALKBH5 in RAW264.7 cells, while overexpression of ALKBH5 reversed this trend (Figure 1B). Given the established involvement of M1 macrophage activation in the pathogenesis of chronic inflammatory diseases including DKD [29], we next investigated whether ALKBH5 could modulate macrophage polarisation in DKD. Flow cytometry showed that ALKBH5 overexpression reduced CD86 expression in LPS/IFN‐γ‐stimulated RAW264.7 macrophages, indicating that M1 polarisation was inhibited (Figure 1C). Conversely, compared with the LPS/IFN‐γ + Vector group, the LPS/IFN‐γ + OE‐ALKBH5 group had increased CD206 expression, further confirming the enhancement of M2 polarisation (Figure 1D). RT‐qPCR analysis demonstrated that ALKBH5 overexpression markedly attenuated LPS/IFN‐γ‐induced upregulation of M1‐associated inflammatory mediators IL‐1β, TNF‐α, and iNOS in RAW264.7 macrophages (Figure 1E). On the other hand, LPS/IFN‐γ stimulation decreased the expression of the M2 marker Arg‐1, while the IL‐10 level showed no significant changes. However, ALKBH5 upregulation promoted the expression of IL‐10 and Arg‐1 under LPS/IFN‐γ conditions (Figure 1F). ELISA analysis confirmed that ALKBH5 augmentation reduced the elevated secretion of pro‐inflammatory cytokines IL‐1β and TNF‐α in RAW264.7 macrophages stimulated with LPS/IFN‐γ (Figure 1G). Compared with the Control group, there was no significant change in the secretion of the anti‐inflammatory cytokine IL‐10 in the LPS/IFN‐γ + Vector group. However, IL‐10 secretion was significantly increased in the LPS/IFN‐γ + OE‐ALKBH5 group compared with the LPS/IFN‐γ + Vector group (Figure 1H).

### 3.2 ALKBH5 Negatively Regulates TRAF6 Through Demethylation Modification

**FIGURE 2:**
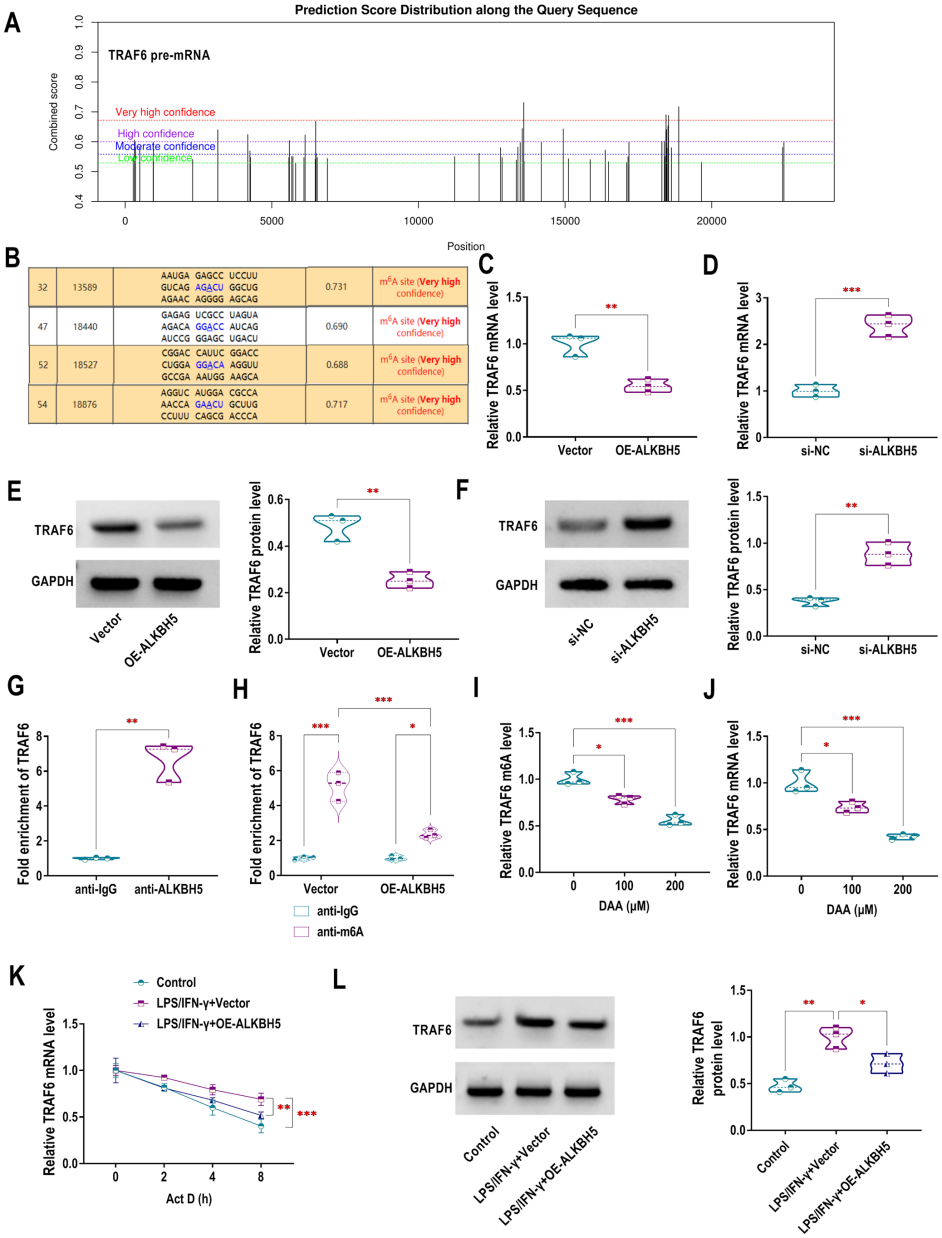
ALKBH5 negatively regulates TRAF6 through demethylation modification. (A, B) The SRAMP database predicted four very high confidence m6A modification sites in TRAF6 mRNA. (C, D) RT‐qPCR was used to quantify TRAF6 mRNA expression in RAW264.7 macrophages after transfection with either Vector/OE‐ALKBH5 (C) or si‐NC/si‐ALKBH5 (D). (E, F) Western blotting was performed to determine TRAF6 protein level following transfection with Vector/OE‐ALKBH5 (E) or si‐NC/si‐ALKBH5 (F). (G) The interaction between ALKBH5 and TRAF6 mRNA was validated using RIP assay. (H) MeRIP assay was applied to assess TRAF6 m6A modification level in ALKBH5‐overexpressing cells. (I, J) The impact of different concentrations of the methylation inhibitor 3‐deazaadenosine (DAA, 0 μM, 100 μM, 200 μM) on TRAF6 m6A modification (I) and mRNA expression (J) was analysed using MeRIP and RT‐qPCR, respectively. (K) TRAF6 mRNA stability in LPS/IFN‐γ‐stimulated RAW264.7 macrophages overexpressing ALKBH5 was evaluated through Act D treatment and RT‐qPCR. (L) Western blotting was used to examine TRAF6 protein expression in LPS/IFN‐γ‐stimulated RAW264.7 macrophages transfected with either Vector or OE‐ALKBH5. *p < 0.05, **p < 0.01, ***p < 0.001.

Next, we explored the downstream target of ALKBH5 affecting DKD progression. SRAMP database analysis predicted four highly confident m6A modification sites in the TRAF6 pre‐mRNA, suggesting that TRAF6 could be modified by m6A (Figure 2A,B). RT‐qPCR (Figure 2C,D) and western blotting (Figure 2E,F) showed that ALKBH5 upregulation reduced TRAF6 expression at both mRNA and protein levels, while ALKBH5 knockdown increased TRAF6 expression. These findings established ALKBH5 as a negative regulator of TRAF6. RIP assay revealed that ALKBH5 directly bound to TRAF6 mRNA (Figure 2G). MeRIP assay further confirmed that ALKBH5 overexpression decreased the m6A modification level of TRAF6 mRNA, indicating that ALKBH5 regulated TRAF6 expression by altering its m6A modification (Figure 2H). Exposure of RAW264.7 macrophages to the methyltransferase inhibitor 3‐deazaadenosine (DAA) significantly attenuated TRAF6 mRNA m6A methylation in a dose‐dependent manner (Figure 2I). After DAA treatment, the expression level of TRAF6 mRNA was also decreased (Figure 2J). ALKBH5 overexpression reversed the LPS/IFN‐γ‐induced suppression of TRAF6 mRNA degradation in RAW264.7 macrophages (Figure 2K). Additionally, OE‐ALKBH5 counteracted the LPS/IFN‐γ‐mediated upregulation of TRAF6 protein expression (Figure 2L).

### 3.3 ALKBH5 Enhances M2 Macrophage Polarisation and Inhibits M1 Polarisation by Negatively Regulating TRAF6

**FIGURE 3:**
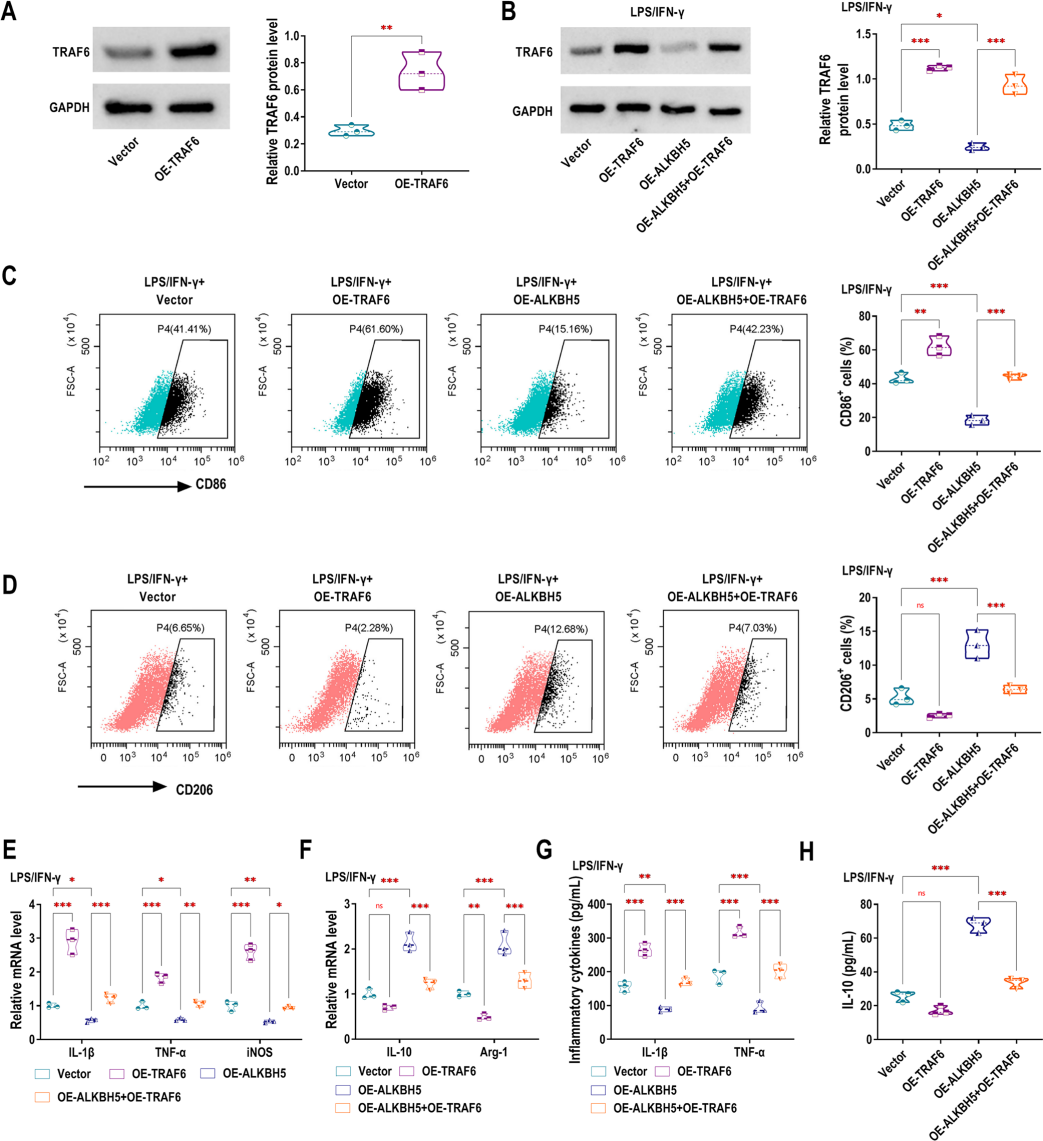
ALKBH5 enhances M2 macrophage polarisation and inhibits M1 polarisation by negatively regulating TRAF6. (A) TRAF6 expression in RAW264.7 cells was assessed by western blotting following transfection with Vector or OE‐TRAF6. (B–H) RAW264.7 cells were transfected with Vector, OE‐TRAF6, OE‐ALKBH5, or OE‐TRAF6 + OE‐ALKBH5 following stimulation with LPS/IFN‐γ. (B) TRAF6 expression in cells was assessed by western blotting. (C, D) The expression of the M1 macrophage marker CD86 and the M2 macrophage marker CD206 was evaluated through flow cytometry. (E, F) RT‐qPCR was used to quantify the mRNA expression levels of IL‐1β, TNF‐α, and iNOS (E), as well as IL‐10 and Arg‐1 (F). (G, H) ELISA was conducted to measure the levels of IL‐1β, TNF‐α and IL‐10 in the cell culture supernatant. ns, no significance, *p < 0.05, **p < 0.01, ***p < 0.001.

Subsequently, TRAF6 was overexpressed in RAW264.7 macrophages. Western blotting analysis confirmed successful TRAF6 overexpression, with significantly higher protein levels in the OE‐TRAF6 group compared to controls (Figure 3A). TRAF6 expression was upregulated by OE‐TRAF6 but downregulated by ALKBH5 overexpression in LPS/IFN‐γ‐stimulated RAW264.7 macrophages. Notably, TRAF6 overexpression rescued the ALKBH5‐mediated downregulation of TRAF6 (Figure 3B). Flow cytometry revealed the reduced M1 marker CD86 and elevated M2 marker CD206 in ALKBH5‐overexpressing macrophages, and these effects were reversed by TRAF6 overexpression (Figure 3C,D). Similarly, TRAF6 overexpression attenuated the ALKBH5‐induced suppression of M1 markers (IL‐1β, TNF‐α, iNOS) and blocked the upregulation of M2 markers IL‐10 and Arg‐1 (Figure 3E,F). ELISA confirmed that ALKBH5 overexpression reduced IL‐1β/TNF‐α and increased IL‐10 secretion in LPS/IFN‐γ‐stimulated macrophages, whereas TRAF6 overexpression reversed these anti‐inflammatory effects (Figure 3G,H). To further explore the regulation of inflammatory signalling by TRAF6, Western blotting analysis was performed to assess the phosphorylation of key pathway components. LPS/IFN‐γ stimulation markedly increased the levels of TRAF6 (Figure S1A,B), p‐STAT1/STAT1 (Figure S1C), and p‐p65 NF‐κB/p65 NF‐κB (Figure S1D) in RAW264.7 macrophages, indicating activation of both the NF‐κB and STAT1 pathways. These effects were reversed by TRAF6 silencing, which significantly reduced the phosphorylation of STAT1 and p65. These findings suggested that TRAF6 promoted macrophage‐mediated inflammation through activation of NF‐κB and STAT1 signalling pathways.

### 3.4 Isolation and Identification of ALKBH5‐Modified UC‐MSCs Exo

**FIGURE 4:**
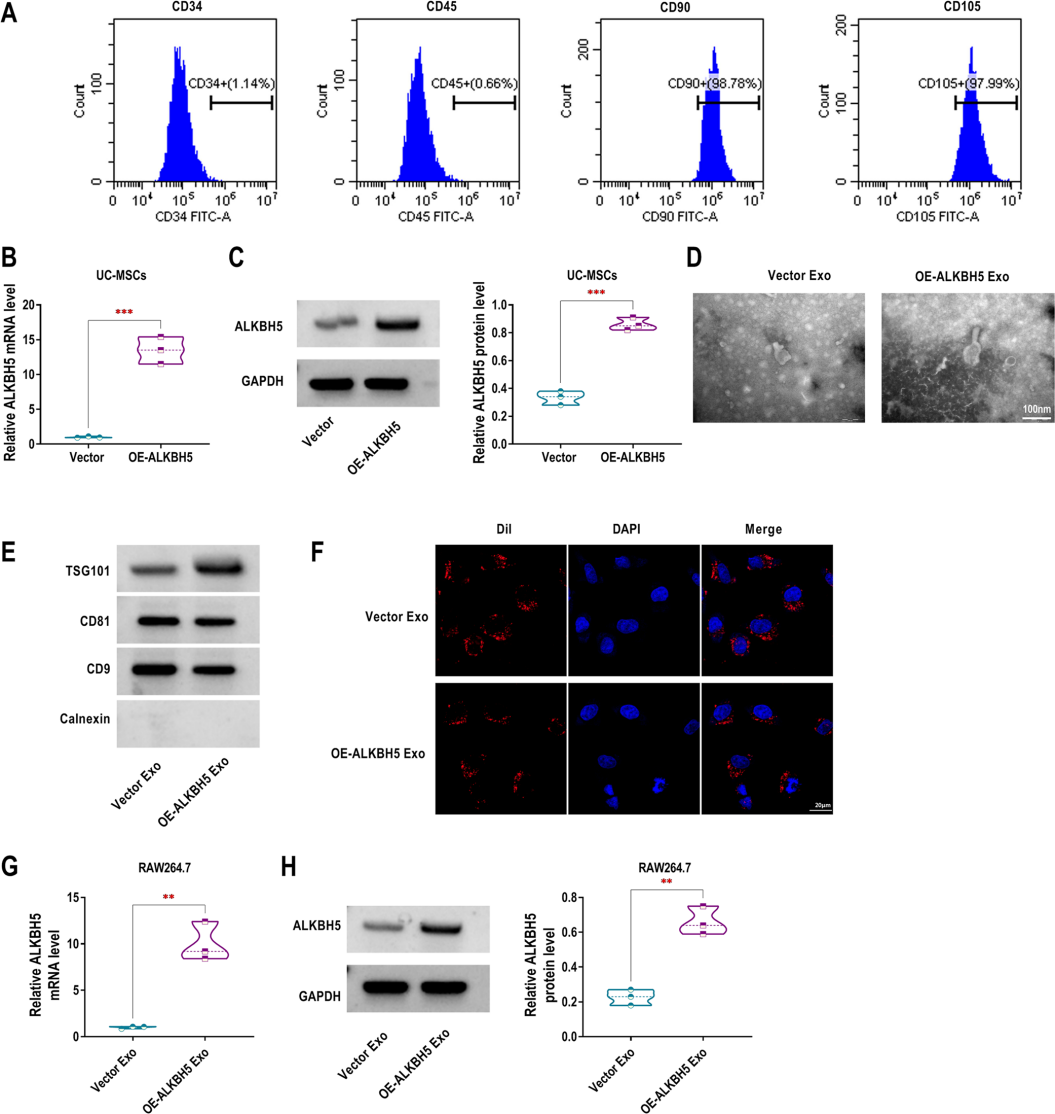
Isolation and Identification of ALKBH5‐modified UC‐MSCs Exo. (A) Specific surface markers of cells were examined by flow cytometry. The UC‐MSCs associated with markers were negative for CD34 and CD45, and were positive for CD90 and CD105. (B, C) RT‐qPCR and western blotting were performed to assess ALKBH5 mRNA and protein expression in UC‐MSCs after OE‐ALKBH5 transfection. (D) TEM images of UC‐MSCs Exo transfected with Vector (Vector Exo) or OE‐ALKBH5 (OE‐ALKBH5 Exo) showed typical spherical morphology, confirming the successful separation. Scale bar, 100 nm. (E) The expression of CD81, CD9 and TSG101 in Vector Exo and OE‐ALKBH5 Exo was detected by western blotting. (F) Fluorescence microscopy showing the uptake of Dil‐labelled Exo by RAW264.7 macrophages after co‐incubation. Scale bar, 20 μm. (G, H) ALKBH5 mRNA and protein expression in RAW264.7 macrophages after co‐incubation with Dil‐labelled UC‐MSCs Exo was measured by RT‐qPCR and western blotting. **p < 0.01, ***p < 0.001.

After obtaining UC‐MSCs from umbilical cords, the expression of UC‐MSCs surface antigens was analysed by flow cytometry. The non‐stem cell markers CD34 and CD45 were expressed at extremely low levels, whereas the stem cell markers CD90 and CD105 were highly expressed (Figure 4A). Transfection of OE‐ALKBH5 in UC‐MSCs promoted the mRNA and protein expression of ALKBH5, confirming the successful overexpression efficiency of OE‐ALKBH5 (Figure 4B,C). After purifying Exo from the culture supernatants of UC‐MSCs, we analysed their morphology and surface markers. TEM images of UC‐MSCs Exo revealed a typical spherical shape, confirming the successful isolation of exosomes (Figure 4D). Western blotting showed that the exosome markers CD81, CD9, and TSG101 were present in UC‐MSCs Exo, while the negative control protein Calnexin was absent, confirming the purity of the Exo (Figure 4E). Dil‐labelled Exo from OE‐ALKBH5 UC‐MSCs were successfully internalised by RAW264.7 macrophages, indicating their cellular uptake (Figure 4F). In addition, the mRNA and protein expression of ALKBH5 in the OE‐ALKBH5 Exo group was higher than that in the Vector Exo group (Figure 4G,H).

### 3.5 ALKBH5‐Modified UC‐MSCs Exo Drive M2 Macrophage Polarisation and Restrain M1 Polarisation

**FIGURE 5:**
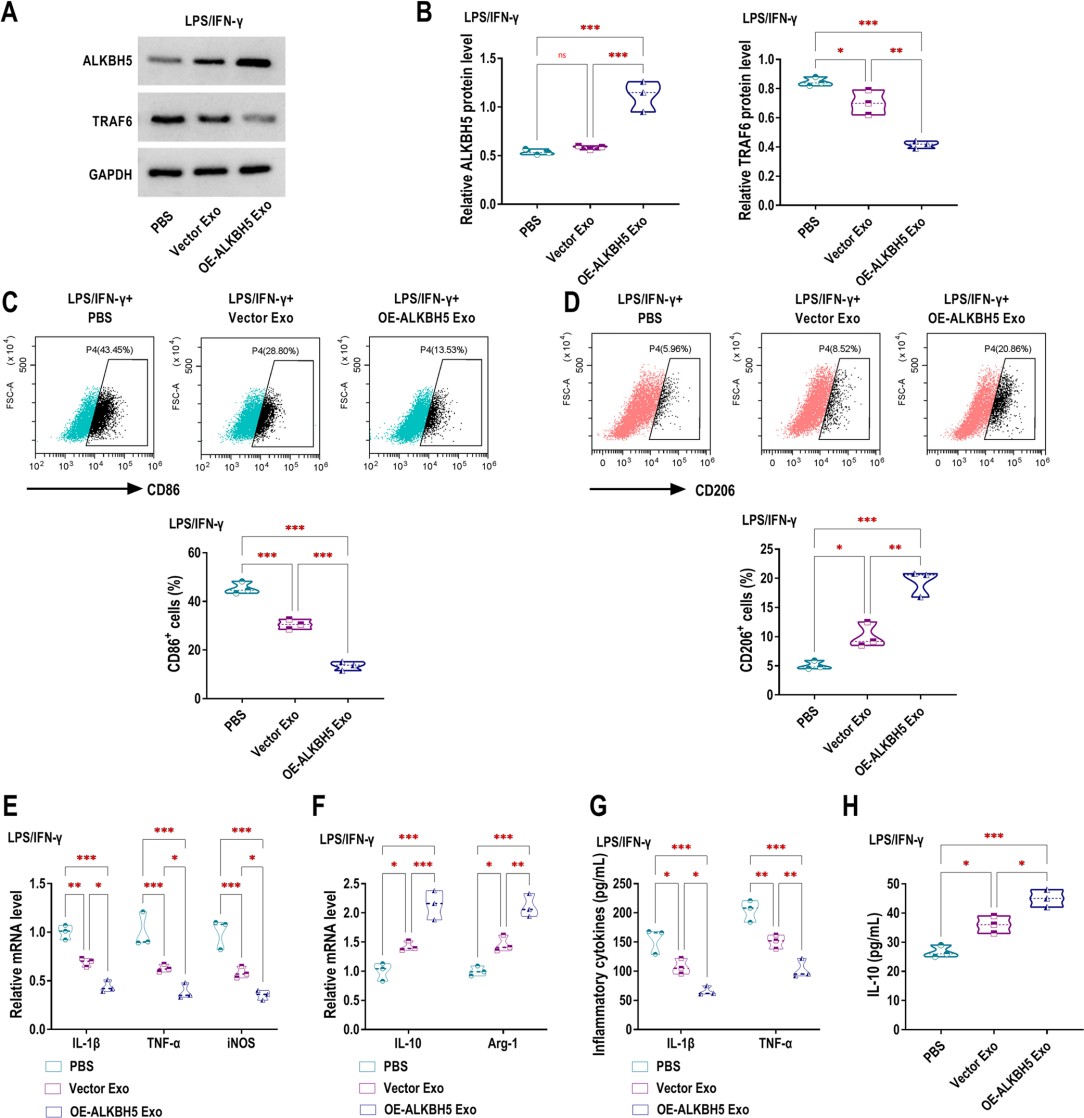
ALKBH5‐modified UC‐MSCs Exo drive M2 macrophage polarisation and restrain M1 polarisation. (A, B) The expression of ALKBH5 and TRAF6 was analysed by western blotting in LPS/IFN‐γ‐induced RAW264.7 macrophages treated with PBS, Vector Exo, or OE‐ALKBH5 Exo. (C, D) Flow cytometry was employed to assess the expression of the M1 macrophage marker CD86 and the M2 macrophage marker CD206. (E, F) RT‐qPCR was performed to measure the mRNA expression levels of IL‐1β, TNF‐α, and iNOS (E), along with IL‐10 and Arg‐1 (F). (G, H) The levels of IL‐1β, TNF‐α, and IL‐10 in the cell culture supernatant were quantified using ELISA. ns, no significance, *p < 0.05, **p < 0.01, ***p < 0.001.

Next, we studied the therapeutic effect of ALKBH5‐modified UC‐MSCs Exo on LPS/IFN‐γ‐induced RAW264.7 macrophage polarisation. Western blotting showed that ALKBH5 protein expression remained unchanged between the PBS and Vector Exo groups but was significantly higher in the OE‐ALKBH5 Exo group. Correspondingly, TRAF6 expression decreased in the Vector Exo group compared to PBS and was further reduced in the OE‐ALKBH5 Exo group (Figure 5A,B). Flow cytometry revealed that CD86 expression declined in the Vector Exo group and was further decreased in the OE‐ALKBH5 Exo group (Figure 5C), while CD206 showed the opposite trend (Figure 5D). Moreover, IL‐1β, TNF‐α, and iNOS levels were downregulated in the Vector Exo group compared to the PBS group, with a further decrease in the OE‐ALKBH5 Exo group (Figure 5E). In contrast, IL‐10 and Arg‐1 were upregulated, with the OE‐ALKBH5 Exo group showing the highest expression (Figure 5F). ELISA results showed similar trends, with IL‐1β and TNF‐α secretion decreasing and IL‐10 secretion increasing, particularly in the OE‐ALKBH5 Exo group (Figure 5G,H). The findings suggested that OE‐ALKBH5 Exo enhanced M2 polarisation and suppressed M1 polarisation by downregulating TRAF6. To further verify the role of TRAF6 in ALKBH5‐modified UC‐MSCs Exo on LPS/IFN‐γ‐induced RAW264.7 macrophage polarisation, a TRAF6 overexpression rescue experiment was performed in RAW264.7 macrophages treated with OE‐ALKBH5‐modified UC‐MSCs Exo. The results showed that TRAF6 overexpression partially reversed the decrease in M1 marker CD86 expression (Figure S2A) and the increase in M2 marker CD206 expression (Figure S2B) induced by OE‐ALKBH5 Exo. Analysis of M1‐associated factors IL‐1β, TNF‐α, and iNOS revealed that TRAF6 overexpression attenuated the inhibitory effect of OE‐ALKBH5 Exo on these pro‐inflammatory mediators (Figure S2C). Correspondingly, the expression levels of M2‐associated factors IL‐10 and Arg‐1 indicated that TRAF6 overexpression partially counteracted the promoting effect of OE‐ALKBH5 Exo on their upregulation (Figure S2D). ELISA analysis further confirmed changes in cytokine secretion, showing that TRAF6 overexpression reduced the OE‐ALKBH5 Exo‐induced decreases in IL‐1β and TNF‐α (Figure S2E) and partially inhibited the increase in IL‐10 secretion (Figure S2F). Collectively, these findings indicated that TRAF6 played a critical regulatory role in ALKBH5‐mediated inhibition of M1 polarisation and promotion of M2 polarisation in macrophages.

### 3.6 ALKBH5‐Modified UC‐MSCs Exo Alleviate Renal Injury in DKD Mice

**FIGURE 6:**
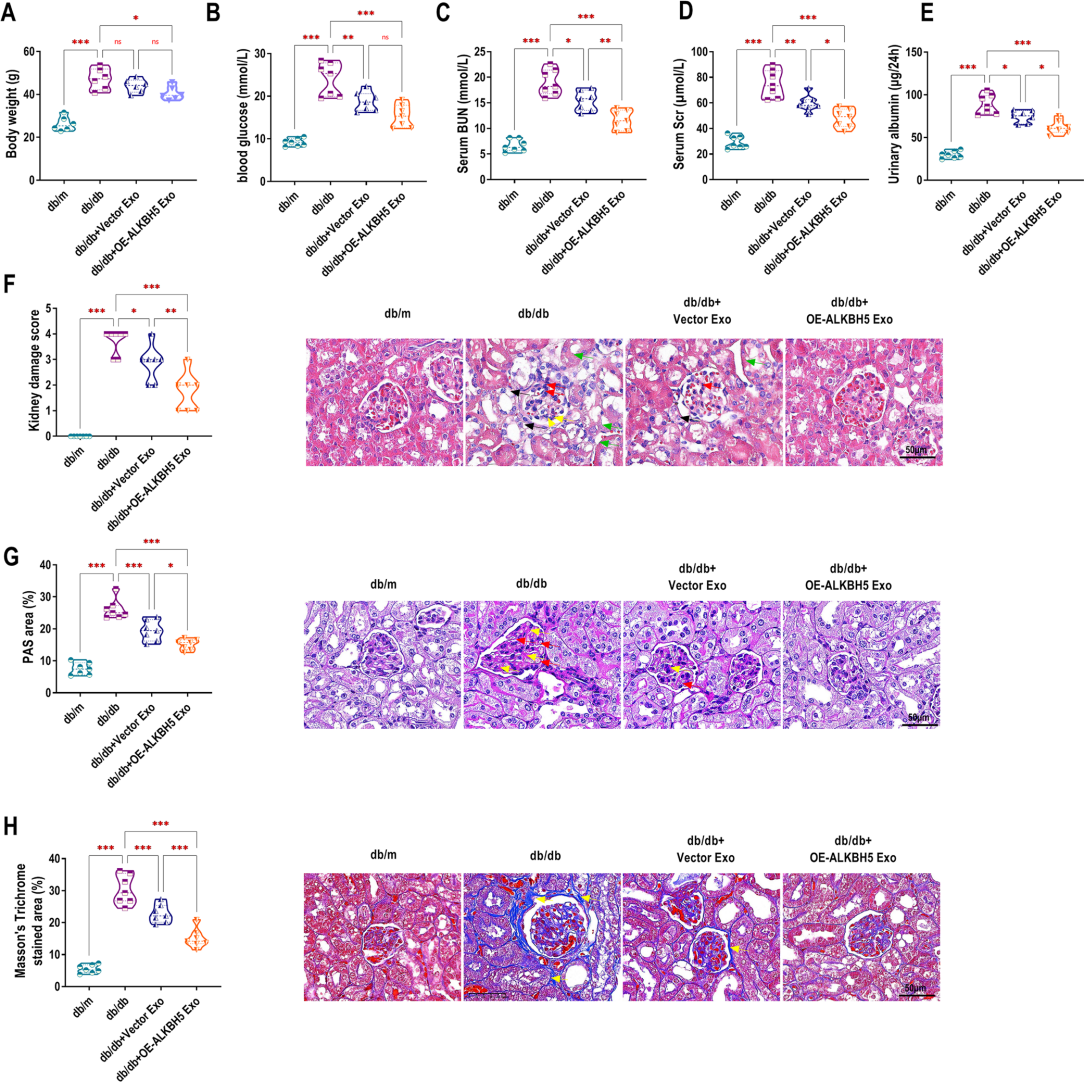
ALKBH5‐modified UC‐MSCs Exo alleviate renal injury in DKD mice. (A–E) Measurement of Body weight (A), blood glucose (B), BUN (C), Scr (D), and 24 h urine albumin (E) levels in control (db/m) and db/db mice treated with Vector Exo or OE‐ALKBH5 Exo. (F) HE staining was used to assess kidney damage, and the damage score was determined. Black arrows: Tubular epithelial cell necrosis, Red arrows: Glomerular mesangial cell proliferation, Yellow arrows: Inflammatory cell infiltration, Green arrows: Thickening of tubular basement membrane. (G) PAS staining was performed to examine tubular damage in kidney sections. Red arrows: Homogeneous thickening of the glomerular basement membrane, Yellow arrows: Increased glomerular mesangial matrix. (H) Masson's trichrome staining was used to evaluate renal fibrosis in the different groups. Yellow arrows: Collagen fibre proliferation. Each group consisted of seven mice. For histological scoring (F–H), five fields per section and three sections per animal were evaluated, and the average was used for analysis. ns, no significance, *p < 0.05, **p < 0.01, ***p < 0.001.

To explore the renal protective effects of ALKBH5‐modified UC‐MSCs Exo in DKD, we injected Vector Exo or OE‐ALKBH5 Exo into the spontaneously diabetic db/db mice (a well‐established DKD model). As shown in Figure 6A, the body weight of db/db mice was significantly higher than that of db/m control. The db/db + OE‐ALKBH5 Exo group showed reduced body weight compared to the db/db group. Blood glucose level was higher in the db/db group compared to the db/m control group. The db/db + Vector Exo group showed a reduction in blood glucose, while the db/db + OE‐ALKBH5 Exo group exhibited no significant difference from the Vector Exo group, but it was lower than the db/db group (Figure 6B). BUN and Scr were key indicators of renal function. In the db/db group, both BUN and Scr levels were significantly elevated. Treatment with Vector Exo resulted in a decrease in BUN and Scr, and further reduction was observed in the db/db + OE‐ALKBH5 Exo group (Figure 6C,D), suggesting an improvement in renal function. Similarly, the 24‐h urinary albumin excretion was highest in the db/db group, decreased in the db/db + Vector Exo group, and further reduced in the db/db + OE‐ALKBH5 Exo group (Figure 6E). HE staining revealed that the kidney damage score was higher in the db/db group. The score was reduced in the db/db + Vector Exo group, and the lowest score was observed in the db/db + OE‐ALKBH5 Exo group (Figure 6F). PAS staining showed more severe tubular damage in the db/db group, which was alleviated by Vector Exo and further improved by OE‐ALKBH5 Exo treatment (Figure 6G). Masson's trichrome staining demonstrated significant renal fibrosis in the db/db group, which was reduced in the db/db + Vector Exo group and further diminished in the db/db + OE‐ALKBH5 Exo group (Figure 6H).

### 3.7 ALKBH5‐Modified UC‐MSCs Exo Strengthen Anti‐Inflammation and Stimulate M2 Macrophage Polarisation in DKD Mice

**FIGURE 7:**
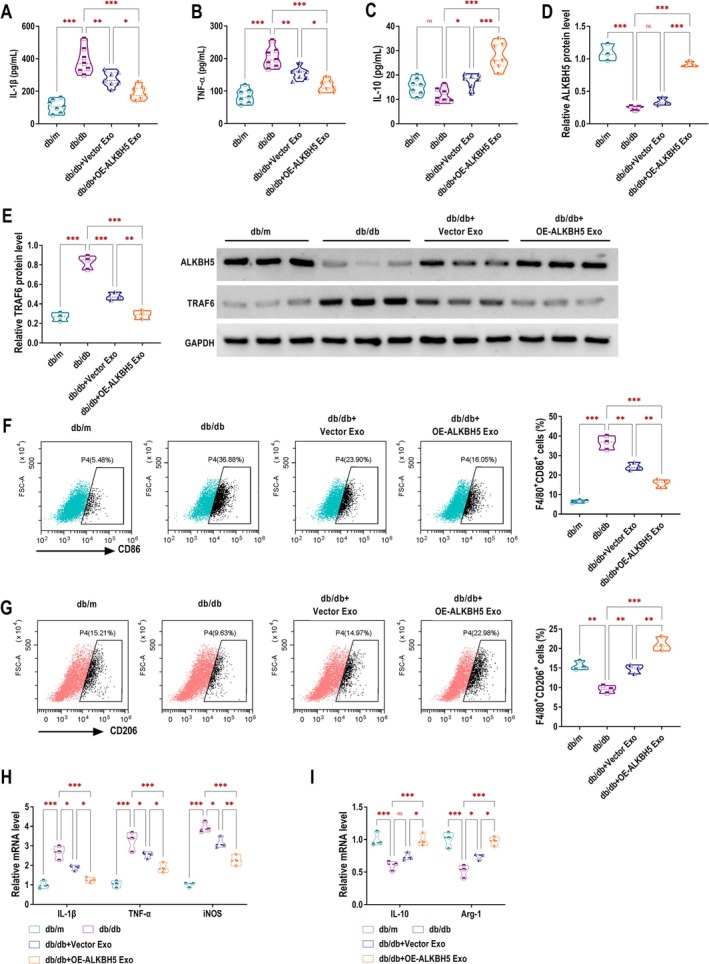
ALKBH5‐modified UC‐MSCs Exo strengthen anti‐inflammation and stimulate M2 macrophage polarisation in DKD mice. (A–C) The serum concentrations of IL‐1β (A), TNF‐α (B), and IL‐10 (C) were measured by ELISA in db/m control mice, as well as in db/db mice treated with either Vector Exo or OE‐ALKBH5 Exo. (D, E) The expression of ALKBH5 and TRAF6 in the four groups of mice was analysed by western blotting. (F, G) Flow cytometry analysis was used to assess the proportion of M1 (F4/80^+^ CD86^+^) and M2 (F4/80^+^ CD206^+^) macrophages in each group. (H, I) The mRNA levels of IL‐1β, TNF‐α, iNOS, IL‐10 and Arg‐1 in each group were detected by RT‐qPCR. ns, no significance, *p < 0.05, **p < 0.01, ***p < 0.001.

Next, the levels of inflammatory factors in the serum of mice were detected by ELISA. The db/db group had higher IL‐1β and TNF‐α levels and no significant change in IL‐10 level compared to the db/m control group. Vector Exo treatment reduced IL‐1β and TNF‐α while increasing IL‐10. The db/db + OE‐ALKBH5 Exo group showed further reduction in IL‐1β (Figure 7A) and TNF‐α (Figure 7B) and an increase in IL‐10 (Figure 7C), indicating reduced inflammation. The db/db group exhibited reduced ALKBH5 expression (Figure 7D) and elevated TRAF6 expression (Figure 7E) compared to the db/m group. In contrast, treatment with Vector Exo led to no change in ALKBH5 expression and a decrease in TRAF6 expression. Notably, the db/db + OE‐ALKBH5 Exo group showed a significant increase in ALKBH5 expression and a marked decrease in TRAF6 level. Flow cytometry analysis showed that the db/db group had more M1 macrophages (F4/80^+^ CD86^+^), while the db/db + Vector Exo group showed fewer M1 macrophages and more M2 macrophages (F4/80^+^ CD206^+^). The db/db + OE‐ALKBH5 Exo group had the highest proportion of M2 macrophages and the lowest M1 macrophages, indicating enhanced M2 polarisation (Figure 7F,G). Compared with the db/db group, both the db/db + Vector Exo and db/db + OE‐ALKBH5 Exo groups exhibited significantly reduced expression levels of IL‐1β, TNF‐α, and iNOS. Moreover, these inflammatory markers were further downregulated in the db/db + OE‐ALKBH5 Exo group compared to the db/db + Vector Exo group (Figure 7H). In addition, IL‐10 expression in the db/db + Vector Exo group showed no significant difference from the db/db group, whereas Arg‐1 was upregulated. Notably, the db/db + OE‐ALKBH5 Exo group (compared to the db/db group) displayed increased expression of both IL‐10 and Arg‐1, which were also higher than those in the db/db + Vector Exo group (Figure 7I). To further confirm the histological alterations of ALKBH5 and TRAF6 in DKD, IHC staining was performed on kidney tissues. As shown in Figure S3, the db/db group exhibited markedly reduced ALKBH5 expression and increased TRAF6 expression compared with the db/m group. Treatment with OE‐ALKBH5 Exo significantly restored ALKBH5 expression and suppressed TRAF6 expression, supporting the potential involvement for the ALKBH5‐TRAF6 regulatory axis in DKD.

## 4 Discussion

DKD affects 20%–40% of diabetes patients and poses significant clinical challenges [30]. Notably, end‐stage renal disease in DKD carries a 20% mortality rate exceeding many cancer‐related deaths [31]. The standard clinical treatment for DKD primarily focuses on strict blood glucose and blood pressure control to slow disease progression. However, despite extensive efforts to manage the condition, many patients still progress from early and mid‐stage DKD to end‐stage chronic kidney disease. Currently, available therapies remain limited, and none can effectively cure DKD [32]. This study demonstrated that ALKBH5 overexpression in UC‐MSC‐derived exosomes attenuated diabetic kidney injury by modulating TRAF6 m6A methylation and promoting M2 macrophage polarisation. Specifically, OE‐ALKBH5 exosomes decreased pro‐inflammatory cytokine levels, improved renal function, reduced histological injury, and enhanced anti‐inflammatory macrophage markers. These findings support the therapeutic potential of ALKBH5‐engineered exosomes in modulating renal inflammation and fibrosis in DKD.

Recent studies have highlighted the critical involvement of macrophages in DKD progression. In DKD, a substantial accumulation of monocytes/macrophages occurs in the glomeruli and tubulointerstitial regions. Emerging evidence suggests that inflammatory cell recruitment and subsequent cytokine production drive both the initiation and aggravation of DKD [33]. Macrophages exhibit functional plasticity, differentiating into either pro‐inflammatory M1 or anti‐inflammatory M2 polarised states. During early kidney injury, M1 macrophages, induced by IFN‐γ and pro‐inflammatory signals, highly express iNOS and CD86, releasing IL‐1β, IL‐6, and TNF‐α to combat infection. In contrast, M2 macrophages, marked by CD206, secrete IL‐10 and Arg‐1, promoting anti‐inflammatory responses and kidney repair [34]. In DKD, M1 macrophages increase podocyte permeability and induce apoptosis, aggravating inflammation, while M2 macrophages are involved in resolution and healing phases [35]. Targeting macrophage polarisation has been demonstrated to reduce renal inflammation and fibrosis in DKD models [36]. Thus, enhancing the differentiation and functional activation of M2‐type macrophages or delivering alternatively activated macrophages and their inducing factors over M1 macrophages holds promise as a therapeutic strategy for DKD.

Emerging evidence implicates m6A RNA methylation in the development of diabetic retinopathy [37]. The m6A epitranscriptomic modification serves as a key regulator of macrophage polarisation states and effector functions [8]. The m6A reader IGF2BP2 facilitates M2 macrophage polarisation and enhances the malignant behaviour of bladder cancer by stabilising NRP1 mRNA expression [38]. ALKBH5, as an m6A demethylase, regulates immune responses in diabetes and obesity [39, 40, 41]. The current study demonstrated that ALKBH5 expression was downregulated in RAW264.7 macrophages activated by LPS and IFN‐γ. Elevated ALKBH5 expression enhanced M2 polarisation and attenuated M1 polarisation, revealing its critical role in macrophage immunomodulation and suggesting potential therapeutic strategies for DKD.

To explore the molecular mechanism of ALKBH5 in DKD, bioinformatics analysis identified potential m6A modification sites on TRAF6 mRNA, suggesting that ALKBH5 might regulate TRAF6 in an m6A‐dependent manner. TRAF6 contains a canonical RING finger domain and belongs to the E3 ubiquitin ligase family, capable of catalysing the formation of polyubiquitin chains. Its signalling activity relies on ubiquitination and interaction with TGF‐β–activated kinase 1 (TAK1), leading to NF‐κB pathway activation [42]. The NF‐κB pathway has been demonstrated to promote macrophage polarisation toward the pro‐inflammatory M1 phenotype and exacerbate inflammation [43]. Importantly, TRAF6 functions as a central adaptor that links upstream receptor signals to downstream inflammatory responses. After activation, TRAF6 mediates ubiquitination to recruit and activate downstream kinase complexes, thereby initiating NF‐κB and MAPK signalling pathways [44, 45]. Persistent activation of these pathways promotes the upregulation of multiple pro‐inflammatory cytokines and exacerbates tissue inflammation and injury. In DKD, TRAF6 has been identified as a key mediator of inflammation and a promising therapeutic target, with its inhibition shown to alleviate renal inflammation in diabetic mice [46]. In our study, RIP and MeRIP assays confirmed that ALKBH5 removed m6A modifications from TRAF6 mRNA, reducing its stability and thereby inhibiting TRAF6 protein expression. Additionally, ALKBH5 negatively regulated TRAF6, promoting M2 macrophage polarisation and suppressing M1 polarisation. Furthermore, TRAF6 knockdown inhibited the activation of both NF‐κB and STAT1 signalling pathways. Consequently, suppression of TRAF6 may represent an effective strategy to mitigate macrophage‐driven inflammation in DKD.

With advancements in regenerative medicine, MSCs particularly those from human umbilical cords, represent a promising avenue for the therapy of DKD treatment. Growing evidence indicates that MSCs‐based therapy can benefit DKD patients by reducing proteinuria [47]. Current research indicates that the therapeutic benefits of MSCs are primarily mediated through exosome‐dependent paracrine signalling [48]. UC‐MSCs Exo demonstrate significant anti‐inflammatory properties via reduction of pro‐inflammatory cytokine levels and suppressing NLRP3 inflammasome activation in podocytes under hyperglycemic conditions, thereby ameliorating renal injury in diabetic mice [49]. The therapeutic application of unmodified MSCs Exo in DKD remains limited due to incomplete characterisation of their bioactive components, which contributes to nonspecific effects and potential off‐target reactions [21]. To address these challenges, strategic engineering of MSCs Exo is required to enhance their therapeutic precision for DKD treatment. Genetic modification of MSCs enables production of therapeutic Exos with enhanced microRNA cargo. Fiore et al. [50] employed an adenoviral system to deliver the human insulin‐like growth factor I (IGF‐I) transgene into UC cord perivascular cells. Their findings revealed that extracellular vesicles (EVs) derived from IGF‐I‐modified MSCs exerted stronger anti‐fibrotic effects in a thioacetamide‐induced liver fibrosis model compared to EV‐depleted conditioned medium. Moreover, the therapeutic efficacy of UC cord MSCs Exo loaded with bone morphogenetic protein 7 was significantly superior to that of unmodified or negative control exosomes in reversing hepatic stellate cell activation and attenuating liver fibrosis [51]. To enhance the efficacy of exosome‐based therapy, we genetically modified UC‐MSCs to overexpress ALKBH5, producing exosomes (OE‐ALKBH5 Exo) enriched with targeted m6A regulatory capacity. It was found that ALKBH5‐modified UC‐MSCs Exo could promote macrophage M2 polarisation and inhibit M1 polarisation, exerting potent renoprotective effects in the DKD mouse model. ADSC‐derived exosomes demonstrate significant renoprotective effects in a DKD model. Jin et al. reported the reduced proteinuria, Scr, and BUN levels, along with attenuated podocyte apoptosis in db/db mice following ADSC‐Exos treatment [26]. Similarly, in STZ‐induced diabetic rats, ADSC‐Exos administration decreased urinary albumin‐to‐creatinine ratio, suppressed mesangial expansion, and ameliorated renal fibrosis [52]. Our results showed that after OE‐ALKBH5 Exo transplantation, blood glucose level remained unchanged, while BUN, Scr, and 24‐h urinary albumin levels were significantly reduced compared to the db/db + Vector Exo group. Imaging analysis revealed that OE‐ALKBH5 Exo treatment significantly alleviated renal vacuolar degeneration, inflammatory cell infiltration, and interstitial fibrosis. These findings suggested that ALKBH5‐modified UC‐MSCs Exo mitigated DKD progression in the murine model. Extensive evidence indicates inflammation as a major factor contributing to DKD progression, with M1/M2 macrophages imbalance exerting a fundamental influence on the inflammatory response [53]. In this study, we demonstrated that ALKBH5‐engineered UC‐MSCs Exo alleviated renal inflammation by promoting macrophage polarisation from the pro‐inflammatory M1 type to the anti‐inflammatory M2 type. One limitation of this study is the exclusive use of male mice. Given the established sex‐based differences in DKD pathophysiology and therapeutic responses, the results and conclusions may not be directly extrapolated to female subjects. Additional studies are needed to validate whether similar mechanisms and therapeutic effects are observed in female models.

In summary, ALKBH5‐modified UC‐MSCs Exo reduced TRAF6 expression through m6A demethylation, thereby promoting M2 macrophage polarisation and suppressing M1 polarisation. These findings provided both a novel methodology and theoretical foundation for enhancing the therapeutic potential and therapeutic effectiveness of UC‐MSCs Exo in treating DKD.

## Author Contributions

Conceptualisation and methodology: Hongmei Liu and Huanhuan Wang. Formal analysis and data curation: Yu Mao and Lige Song. Validation and investigation: Lige Song and Zhiqiang Kang. Writing – original draft preparation and writing – review and editing: Lei Li, Hongmei Liu and Huanhuan Wang. Approval of final manuscript: all authors.

## Funding

This study was supported by Medical Research Project of Zhengzhou City in 2024: ZZYK2024033.

## Ethics Statement

The present study was approved by the ethical review committee of Zhengzhou Central Hospital affiliated with Zhengzhou University.

## Consent

Written informed consent was obtained from all enrolled patients.

## Conflicts of Interest

The authors declare no conflicts of interest.

## Supporting information


**Figure S1:** TRAF6 knockdown inhibits NF‐κB and STAT1 signalling activation in LPS/IFN‐γ‐stimulated RAW264.7 macrophages. (A–D) RAW264.7 cells stimulated by LPS/IFN‐γ were transfected with si‐NC or si‐TRAF6. The expression of TRAF6, p‐STAT1/STAT1 and p‐p65 NF‐κB/p65 NF‐κB were detected by Western blotting. ***p < 0.001.

**Figure S2:** TRAF6 overexpression reverses ALKBH5‐mediated macrophage polarisation induced by UC‐MSCs Exo. (A, B) Flow cytometry was employed to assess the expression of the M1 macrophage marker CD86 and the M2 macrophage marker CD206 in LPS/IFN‐γ‐induced RAW264.7 macrophages treated with Vector Exo, OE‐ALKBH5 Exo, or OE‐ALKBH5 Exo + OE‐TRAF6. (C, D) RT‐qPCR was performed to measure the mRNA expression levels of IL‐1β, TNF‐α, and iNOS (C), along with IL‐10 and Arg‐1 (D). (E, F) The levels of IL‐1β, TNF‐α, and IL‐10 in the cell culture supernatant were quantified using ELISA. *p < 0.05, **p < 0.01, ***p < 0.001.

**Figure S3:** IHC analysis of ALKBH5 and TRAF6 expression in kidney tissues from DKD mice. Representative IHC images showing ALKBH5 and TRAF6 expression in kidney sections from db/m, db/db, db/db + Vector Exo, and db/db + OE‐ALKBH5 Exo groups.

**Table S1:** The primers used for RT‐qPCR.

**Table S2:** Metabolic and Physiological Parameters of db/db Mice Before and After Modelling.

## Data Availability

The analysed data sets generated during the present study are available from the corresponding author on reasonable request.
