# Supplementary figures and images for: Diabetic Kidney Disease Progression Alleviated in Mice by ALKBH5‐Mediated UC‐MSCs‐Derived Exosomes That Inhibit TRAF6 m6A Modification and Promote M2 Macrophage Polarisation
Source: Endocrinol Diabetes Metab. 2026 Jan 13;9(1):e70131. doi: 10.1002/edm2.70131 (PMC12796834; doi:10.1002/edm2.70131)

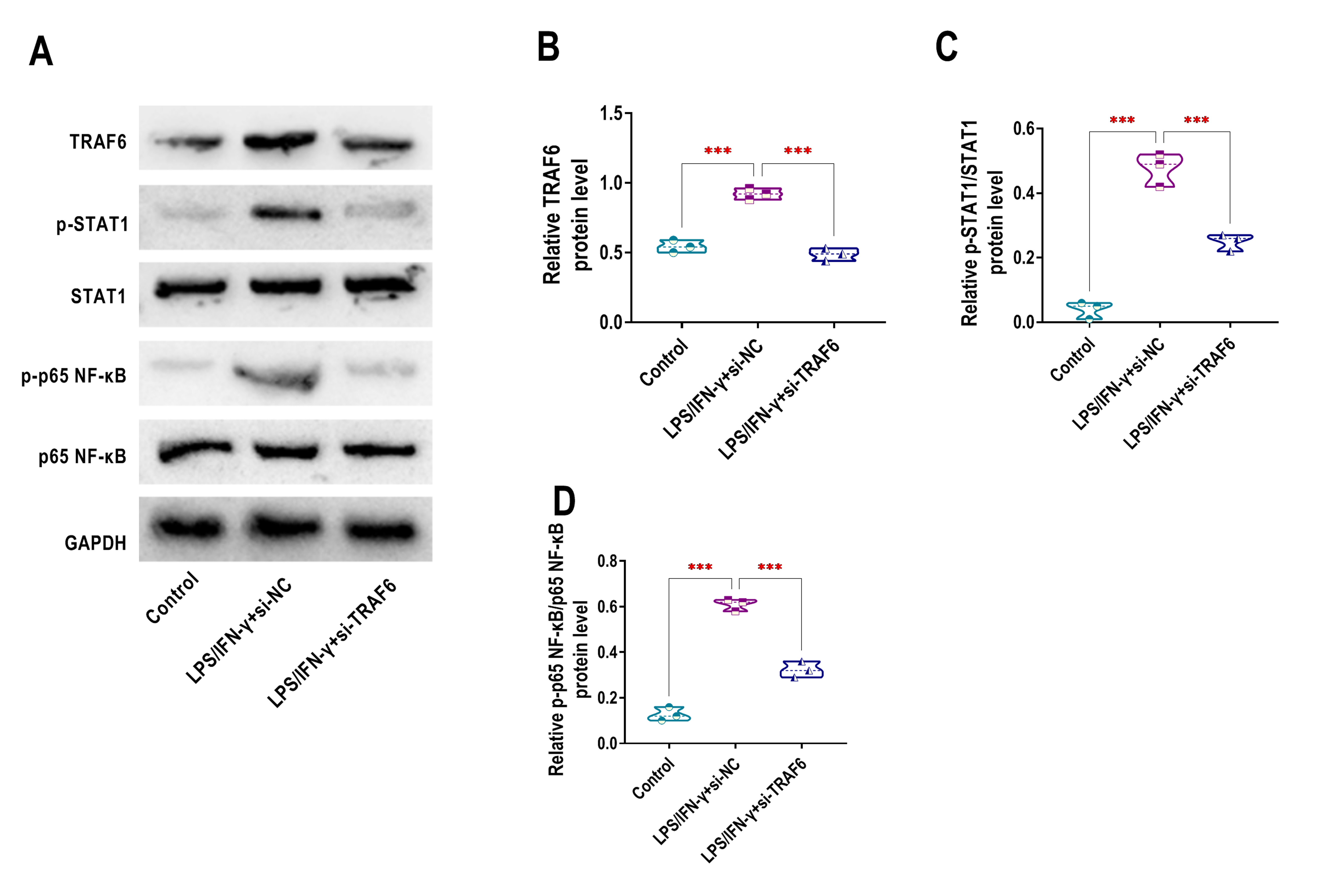

Supplement: Supplementary file 1 — Figure S1: TRAF6 knockdown inhibits NF‐κB and STAT1 signalling activation in LPS/IFN‐γ‐stimulated RAW264.7 macrophages. (A–D) RAW264.7 cells stimulated by LPS/IFN‐γ were transfected with si‐NC or si‐TRAF6. The expression of TRAF6, p‐STAT1/STAT1 and p‐p65 NF‐κB/p65 NF‐κB were detected by Western blotting. ***p < 0.001. [file EDM2-9-e70131-s002.tif]

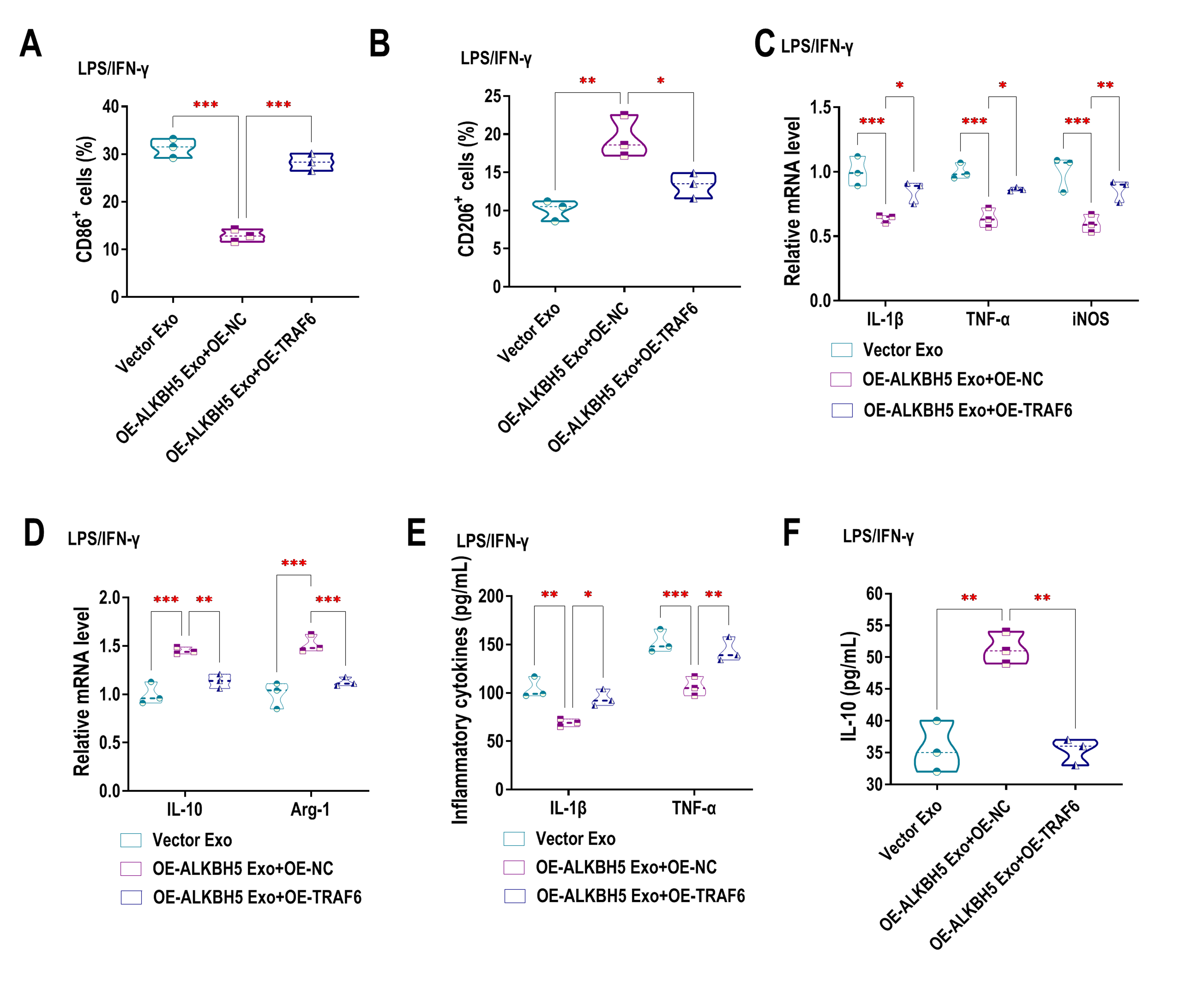

Supplement: Supplementary file 2 — Figure S2: TRAF6 overexpression reverses ALKBH5‐mediated macrophage polarisation induced by UC‐MSCs Exo. (A, B) Flow cytometry was employed to assess the expression of the M1 macrophage marker CD86 and the M2 macrophage marker CD206 in LPS/IFN‐γ‐induced RAW264.7 macrophages treated with Vector Exo, OE‐ALKBH5 Exo, or OE‐ALKBH5 Exo + OE‐TRAF6. (C, D) RT‐qPCR was performed to measure the mRNA expression levels of IL‐1β, TNF‐α, and iNOS (C), along with IL‐10 and Arg‐1 (D). (E, F) The levels of IL‐1β, TNF‐α, and IL‐10 in the cell culture supernatant were quantified using ELISA. *p < 0.05, **p < 0.01, ***p < 0.001. [file EDM2-9-e70131-s003.tif]

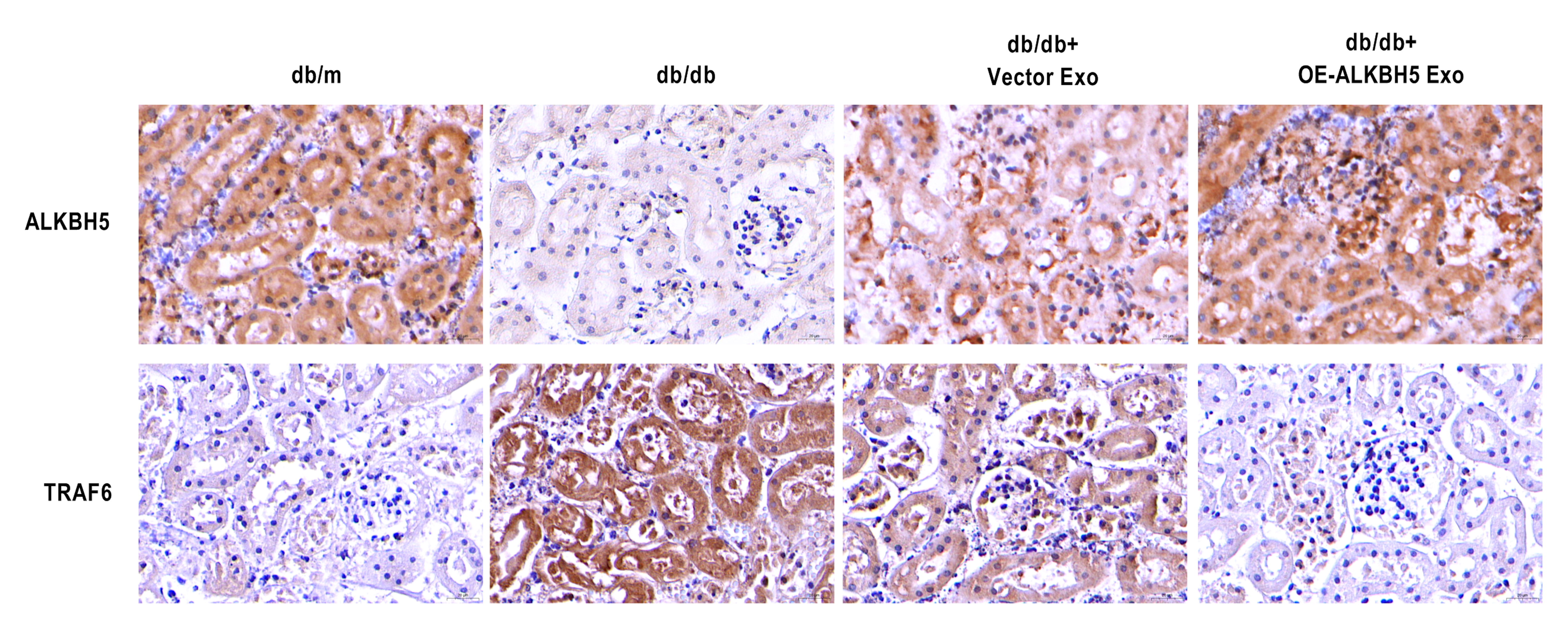

Supplement: Supplementary file 3 — Figure S3: IHC analysis of ALKBH5 and TRAF6 expression in kidney tissues from DKD mice. Representative IHC images showing ALKBH5 and TRAF6 expression in kidney sections from db/m, db/db, db/db + Vector Exo, and db/db + OE‐ALKBH5 Exo groups. [file EDM2-9-e70131-s001.tif]
