# Supplementary material for: Diabetic Kidney Disease Progression Alleviated in Mice by ALKBH5‐Mediated UC‐MSCs‐Derived Exosomes That Inhibit TRAF6 m6A Modification and Promote M2 Macrophage Polarisation
Source: Endocrinol Diabetes Metab. 2026 Jan 13;9(1):e70131. doi: 10.1002/edm2.70131 (PMC12796834; doi:10.1002/edm2.70131)
Supplement: Supplementary file 4 — Table S1: The primers used for RT‐qPCR. [file EDM2-9-e70131-s004.docx]

**Table S1.** **The primers used for RT-qPCR**

| Primer name | Primer sequence (5’-3’) |
| --- | --- |
| iNOS-F | CAGGAGGAGAGAGATCCGATTTA |
| iNOS-R | GCATTAGCATGGAAGCAAAGA |
| IL-1β-F | TGCCACCTTTTGACAGTGATG |
| IL-1β-R | ATGTGCTGCTGCGAGATTTG |
| TNF-α-F | ATGGCCTCCCTCTCATCAGT |
| TNF-α-R | TTTGCTACGACGTGGGCTAC |
| IL-10-F | CTGGACAACATACTGCTAACCG |
| IL-10-R | GGGCATCACTTCTACCAGGTAA |
| Arg-1-F | TGTCCCTAATGACAGCTCCTT |
| Arg-1-R | GCATCCACCCAAATGACACAT |
| TRAF6-F | TAAGGGATGCAGGGCACAAG |
| TRAF6-R | GGCACTTTACCGTCAGGGAA |
| ALKBH5-F | ACGTTGACCCCATCCACATC |
| ALKBH5-R | AATGTCCTGAGGCCGTATGC |
| GAPDH-F | AGGTCGGTGTGAACGGATTTG |
| GAPDH-R | TGTAGACCATGTAGTTGAGGTCA |
