# Supplementary material for: Diabetic Kidney Disease Progression Alleviated in Mice by ALKBH5‐Mediated UC‐MSCs‐Derived Exosomes That Inhibit TRAF6 m6A Modification and Promote M2 Macrophage Polarisation
Source: Endocrinol Diabetes Metab. 2026 Jan 13;9(1):e70131. doi: 10.1002/edm2.70131 (PMC12796834; doi:10.1002/edm2.70131)
Supplement: Supplementary file 5 — Table S2: Metabolic and Physiological Parameters of db/db Mice Before and After Modelling. [file EDM2-9-e70131-s005.docx]

**Table S2. Metabolic and Physiological Parameters of db/db Mice Before and After Modeling**

| Parameter | db/db (Pre) | db/db (Post) | *P* value | Success Criteria |
| --- | --- | --- | --- | --- |
| Activity Level | Normal | Reduced | < 0.01 | Significant decrease |
| Coat Condition | Smooth, glossy | Rough, dull | < 0.05 | Visible deterioration |
| Food Intake (g/day) | 3.6 ± 0.3 | 7.5 ± 0.8 | <0.001 | >50% increase |
| Water Intake (mL/day) | 5.1 ± 0.5 | 15.8 ± 2.5 | <0.001 | >2-fold increase |
| Urine Volume (mL/24h) | 1.3 ± 0.2 | 5.9 ± 1.1 | <0.001 | >3-fold increase |
| Fasting Glucose (mmol/L) | 6.2 ± 0.7 | 23.7 ± 3.9 | <0.001 | ≥16.7 mmol/L |
| 24h Urinary Albumin (μg) | 17.3 ± 8.6 | 89.7 ± 12.5 | <0.001 | >3-fold increase |

**Data presented as mean ± SD (n=21)**
